# Copper metabolism in hepatocellular carcinoma: from molecular mechanisms to therapeutic opportunities

**DOI:** 10.3389/fmolb.2025.1578693

**Published:** 2025-05-13

**Authors:** Ziling Pang

**Affiliations:** Department of Nursing, School of Medicine, Shihezi University, Shihezi, China

**Keywords:** copper, hepatocellular carcinoma, copper homeostasis, copper metabolism, cancer therapy

## Abstract

Copper is a vital trace metal that facilitates cell proliferation, angiogenesis, and tumour spread. The liver is essential for copper metabolism, hence regulating copper levels is crucial for hepatic health. Hepatocellular carcinoma is a primary liver cancer characterised by a high death rate, and extensive research has shown the substantial impact of copper on its progression. This research primarily examines the molecular mechanisms involved, summarises the regulation of copper homeostasis, and addresses the role of copper metabolism in the promotion and inhibition of hepatocellular carcinoma development. Furthermore, it investigates prospective clinical approaches for targeting copper in the treatment of this disease, intending to establish a theoretical basis for the clinical use of copper in the management of hepatocellular carcinoma.

## 1 Introduction

Liver cancer ranks as the sixth most diagnosed malignancy and third leading cause of cancer deaths worldwide (Rumgay et al., 2022). Hepatocellular carcinoma (HCC), accounting for 75%–85% of primary liver cancers, poses substantial healthcare challenges (Vogel et al., 2022; Barcena-Varela et al., 2024). The Asia-Pacific region bears 75% of the global HCC burden, with incidence rates continuing to rise (Mak et al., 2024). Emerging evidence highlights copper metabolism as a critical regulator of HCC pathogenesis, offering potential therapeutic avenues.

Copper serves as a fundamental element in cancer biology, functioning as a redox agent that is integral to both normal physiological and pathological processes (Wang et al., 2023; Tsvetkov et al., 2022; Tang et al., 2024). The physiological regulation of copper in the body is a complex process, with the liver principally responsible for its metabolism and storage. Copper absorption relies on many transporter proteins, including copper transporter 1 (CTR1) and divalent metal-ion transporter-1 (DMT1) (Li, 2020; Shawki et al., 2015). Upon cellular entry, copper is distributed to various organelles to fulfil cellular requirements while excess copper is chelated by antioxidant peptides such as metallothionein (MT) and glutathione (GSH) to avert cellular harm caused by reactive oxygen species (ROS) generated during this process (Xue et al., 2023). Copper efflux mostly relies on copper-transporting ATPases, specifically ATPase copper transporting alpha (ATP7A) and ATPase copper transporting beta (ATP7B). When intracellular copper levels are excessively elevated, these transport proteins expel copper from the cell to preserve copper homeostasis (La Fontaine and Mercer, 2007). While essential for energy metabolism and signaling (Xie et al., 2022), copper excess generates ROS that drive oncogenic transformation (Klaunig et al., 2011; Porcu et al., 2018). Aberrant *in vivo* copper metabolism can either facilitate or impede tumorigenesis. Simultaneously, the knockdown of CTR1 or copper chelation diminishes the expression of glycolytic genes and the utilization of downstream metabolites, impeding hepatocellular carcinoma metabolism (Davis et al., 2020). Copper also affects carcinogenesis and progression via immune-related mechanisms (Wu et al., 2023). Consequently, copper absorption, utilization, and outflow are synchronised through various processes to guarantee accurate regulation of intracellular copper levels.

Copper’s biological functions are crucial in the development and progression of HCC, encompassing mechanisms such as tumour cell proliferation, metastasis, and angiogenesis. Research indicates that increased copper concentrations are significantly linked to augmented tumour proliferation and invasiveness, with increasing extracellular copper facilitating HCC cell proliferation, migration, and invasion through modulation of the MYC/CTR1 axis (Porcu et al., 2018). This may pertain to the stimulation of many oncogenic signaling pathways by copper and its provision of energy to HCC cells. Copper may facilitate tumour cell proliferation and viability by stimulating the PI3K/mTOR signaling pathway (Asati et al., 2016). Moreover, copper may augment the adaptive capacity and survival of tumour cells by promoting autophagy, increasing the activity of transcription factors, influencing tumour proteins, and modulating immune-related responses.

Nonetheless, copper may also impede the progression of HCC by an novel cell death process known as cuproptosis. Copper toxicity, associated with mitochondrial malfunction and oxidative stress, has been shown to induce apoptosis in HCC cells, potentially via the enhancement of ROS production and the downregulation of anti-apoptotic protein expression (Xie et al., 2023). Moreover, copper’s impacts on the immune system and the modification of signaling pathways can influence the suppression of HCC. At present, copper chelators and copper ionophore, such as DSF (Disulfiram)/Cu, have demonstrated efficacy in inhibiting the proliferation and viability of HCC cells (Ren et al., 2021). DSF/Cu complex promotes copper death by increasing the concentration of intracellular copper ions. Therefore, DSF/Cu has potential application value in inducing copper death in cancer cells (Ishida et al., 2013a).

The dual role of copper offers novel insights for therapeutic options in the treatment of HCC. Modulating copper levels or targeting signaling pathways related to copper metabolism may yield novel therapy options for HCC patients. Furthermore, triggering copper death may emerge as a novel method for hepatocellular carcinoma treatment, particularly in modulating the tumour immunological milieu and affecting patient prognosis. This article delineates the pivotal function of copper in the aetiology of HCC. It examines the therapeutic justification of copper in HCC within current research and its prospective role in enhancing targeted therapy for HCC.

## 2 Physiological regulation of copper

The liver is the primary organ responsible for the metabolism and storage of copper in the body. Upon ingestion, copper in food binds to serum albumin in the bloodstream; the majority is excreted via the biliary pathway, while a minor fraction is stored and absorbed primarily through the duodenum and small intestine into the liver, where it associates with ceruloplasmin (CP), an essential protein. CP subsequently facilitates the mobilisation of copper into systemic circulation to meet the physiological demands of the body (Kim et al., 2010; An et al., 2022; Yang L. et al., 2023; Jiang et al., 2022).

**FIGURE 1:**
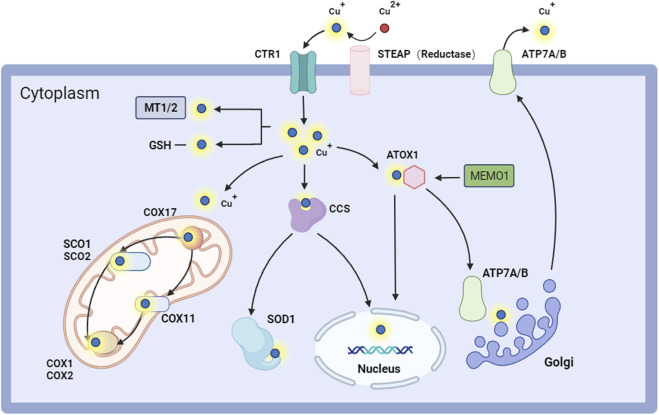
Intracellular copper regulates pathways. STEAP converts Cu^2+^ to Cu^+^ and facilitates its entry into the cell through CTR1. Copper can attach to MT1-2/GSH within the cell, be transported to COX11 and SCO1/2 in the mitochondria by COX17 for cellular respiration, or be directed to SOD1 via CCS for antioxidant activity. Copper ions can penetrate the nucleus via ATOX1 and CCS, influencing gene expression. They can access the Golgi through ATP7A/B to engage in protein glycosylation and other modification activities, thereby maintaining intracellular copper homeostasis and proper functionality. Copper ions are ultimately exported through ATP7A/B. Abbreviation: ATOX1, antioxidant 1 copper chaperone; ATP7A/B, ATPase copper taransporting alpha/beta; CCS, copper chaperone for superoxide dismutase; COX1, cytochrome c oxidase 1; COX11, cytochrome c oxidase 11; COX17, cytochrome c oxidase 17; COX2, cytochrome c oxidase 2; CTR1, copper transporter 1; GSH, glutathione; MEMO1, mediator of cell motility 1; MT1/2, metallothionein1/2; SCO1, synthesis of cytochrome c oxidase 1; SCO1, synthesis of cytochrome c oxidase 2; SOD1, superoxide dismutase 1.

The intricate equilibrium between copper absorption and excretion regulates intracellular copper homeostasis (Royer and Sharman, 2024). The human body regulates copper levels within a specific and limited range, with a U-shaped dose-response curve illustrating the correlation between levels and effects (Stern et al., 2007; Lelièvre et al., 2020). The human body generally stores from 50 to 120 mg of copper, and serum copper levels in healthy people remain steady between 70 and 110 mg/dL, with the top limit of the standard established at approximately 1.5 mg Cu/L. The established safe daily copper intake for adults is 10 mg for women and 12 mg for men, as found by research on health maintenance. The minimum necessary need is established at 0.6–0.7 mg/d, while optimal copper homeostasis is approximately 2.6 mg/d (Wu et al., 2023; Charkiewicz, 2024; Ciosek et al., 2023; Guan et al., 2023). Alongside its association with proteins, a portion of copper in the body exists in a free state, and an imbalance in the concentration of free copper within cells can adversely affect the organism (Gao et al., 2023). Excessive intracellular copper accumulation may result in copper binding to mitochondrial proteins, thereby initiating apoptosis through the formation of ROS and the activation of the mitochondrial fission protein dynamin-related protein 1 (DRP1). Moreover, copper deprivation exacerbates the misfolding of superoxide dismutase 1 (SOD1) and modifies its hydrophobic properties, leading to cellular damage and potential mortality (Liu et al., 2023). Copper homeostasis in the body is regulated by meticulous regulation of dietary intake and intricate regulatory mechanisms within the organism (Figure 1).

### 2.1 Copper uptake

The human body has complex copper transport and copper-binding proteins Table 1 to control copper uptake, intracellular movement, storage, and efflux while effectively curbing copper-free-floating in the cytoplasm to avoid potentially deleterious effects (Shanbhag et al., 2021). In watery conditions, such as bodily fluids, copper predominantly exists in the oxidised state of Cu^2+^. Conversely, upon traversing the cell membrane and entering the intracellular reducing milieu, copper is transformed into its reduced form, Cu^+^, which is essential for cellular absorption and utilization (Lelièvre et al., 2020; Guan et al., 2023; Zhou et al., 2023). Dietary copper predominantly exists as Cu^2+^, necessitating its reduction to Cu^+^ by the STEAP (reductase) family of metal reductases before cellular transit by CTR1 (Li, 2020; Yang D. et al., 2023). CTR1 is a high-affinity copper transmembrane transporter protein at the plasma membrane that operates independently of adenosine triphosphate (ATP). It facilitates the transport of extracellular copper into the cell through specific pathways, aiding in the maintenance of optimal copper concentration within the cell (van den Berghe et al., 2007; Das et al., 2022). The low-affinity homolog copper transporter 2 (CTR2) is responsible for localisation and facilitating translocation from the vesicular compartment into the cytoplasm (Davis et al., 2020). Cluster of differentiation 44 (CD44), present on the cell surface, significantly contributes to metal uptake, particularly in facilitating copper absorption (Solier et al., 2023). Furthermore, the present work elucidates various mechanisms of copper absorption, including DMT1. Oxidised Cu^2+^ is absorbed by DMT-1 (Shawki et al., 2015). Studies demonstrate a substantial negative association between the expression levels of DMT1 and CTR1. Moreover, environments with elevated copper levels can significantly affect the expression pattern of DMT1 in Caco-2 cells, commonly utilised in intestine research, as well as in human umbilical vein endothelial cells (HUVEC) (Ilyechova et al., 2019; Sharp, 2003; Lin et al., 2015). Beyond the established channels, additional processes of copper absorption by intestinal cells may exist that are inadequately understood and necessitate further comprehensive investigation.

**TABLE 1:** Important copper transport proteins.

Name	Abbreviation	Role	References
CTR1	Copper transporter	It is responsible for transporting copper across the membrane and selective uptake of Cu^+^, which is a prerequisite for copper to enter the cell for metabolism.	Sharp (2003), Lin et al. (2015)
COX17	Cytochrome c oxidase copper chaperone 17	It transports intracellular Cu^+^ to mitochondria for loading into cytochrome oxidase.	Maxfield et al. (2004), Zhu et al. (2023)
CCS	Copper chaperone for superoxide dismutase	It delivers Cu^+^ specifically to SOD1 to protect cells from ROS damage.	Grasso et al. (2021), Casareno et al. (1998)
ATOX1	Antioxidant 1 copper chaperone	It acts as a copper chaperone to capture and transfer Cu^+^ to the atpase copper transporter 7A/7B in the trans-Golgi apparatus.	Hatori and Lutsenko (2013), Hamza et al. (2001), Zhang et al. (2022), Beaino et al. (2014)
ATP7A	ATPase copper taransporting alpha	It is expressed in extrahepatic tissues to export copper from the intestine into the blood for further distribution into tissues.	Shao et al. (2023), Horn and Wittung-Stafshede (2021), Lukanović et al. (2020)
ATP7B	ATPase copper transporting beta	It is expressed in the liver, exporting copper from the liver to the bile, and is associated with copper overload.	Lukanović et al. (2020)
MT	Metallothionein	It is a low-molecular-weight, cysteine-rich metal-binding protein that chelates copper and other metals from the body.	Shawki et al. (2015), Ilyechova et al. (2019)

### 2.2 Copper utilization

Copper entering the cell is swiftly distributed to different cellular regions to meet diverse cellular requirements. While bonded unstable copper can produce ROS or induce cytotoxicity in cells, surplus intracellular copper can be chelated by two principal antioxidant peptides: MT and GSH (Tang et al., 2024; Freedman et al., 1989; Calvo et al., 2017). This mechanism depends on the collaborative function of many copper-transporting and chaperone proteins. Upon cellular entry, a portion of copper is sequestered by MT, which serves as an efficient reservoir that chelates and retains surplus intracellular copper. Additionally, MT functions as an antioxidant by inhibiting the redox cycle of copper, thereby diminishing the production of ROS and effectively safeguarding against copper-induced oxidative stress, thus preserving cellular structural and functional integrity (Behar and Maayan, 2023; Luza and Speisky, 1996; Mosna et al., 2023). Copper is delivered to subcellular organelles for bioavailability in cells by many co-conjugated proteins, including cytochrome c oxidase copper chaperone 17 (COX17), Copper chaperone for superoxide dismutase (CCS), and antioxidant 1 copper chaperone (ATOX1).

COX17 is a crucial protein for the assembly of cytochrome c oxidase in mitochondria, functioning exclusively within the mitochondrial membrane space, where it facilitates copper transport to the respiratory chain and mediates copper exocytosis from the mitochondria. Initially, copper traverses the outer mitochondrial membrane through COX17, thereafter passing through the inner mitochondrial membrane into the mitochondrial matrix via solute carrier family 25 member 3 (SLC25A3) (Murata et al., 2024). COX17 is implicated in copper transport to cytochrome c oxidase (CCO) and may potentially participate in copper efflux from mitochondria (Maxfield et al., 2004; Wang et al., 2013; Zhu et al., 2023).

CCS serves as the sole copper chaperone protein for CuZn-SOD1, a principal cytoplasmic antioxidant enzyme and a prospective anti-cancer target (Grasso et al., 2021). It activates SOD1 to sustain intracellular redox homeostasis and facilitates the effective transport of copper to mitochondria, thereby positively impacting the functionality of copper proteins and mitochondrial energy metabolism (Casareno et al., 1998). CCS does not disrupt the standard transfer of copper to the CCO while promoting copper transport to the mitochondria, hence ensuring accurate distribution and utilization of copper among many intracellular targets (Wang et al., 2013; Wang et al., 2021a). Furthermore, copper is transported by the CCS to the nucleus, where it activates the transcription factor hypoxia-inducible factor 1 (HIF1) (Feng et al., 2009).

ATOX1 is integral to the copper metabolic pathway (Yang D. et al., 2023). It can sequester and convey copper to ATP7A/7B inside the trans golgi network (TGN) for effective copper transport and distribution (Xue et al., 2023; Hatori and Lutsenko, 2013; Hamza et al., 2001). The protein mediator of ERBB2-driven cell motility 1 (MEMO1) augmented the binding affinity of ATOX1 to Cu^+^, a mechanism that mitigates Cu-induced ROS overproduction, thus safeguarding cells from oxidative stress damage (Zhang et al., 2022). Moreover, ATOX1 plays a crucial role in the synthesis of copper-dependent enzymes, including CP and lysyl oxidase (LOX), which are vital for preserving intracellular copper homeostasis and the functionality of these critical enzymes (Blockhuys et al., 2020). ATOX1 facilitates the translocation of copper into the nucleus and functions as a copper-dependent transcription factor (Beaino et al., 2014; Itoh et al., 2008).

### 2.3 Copper export

Excessive copper content within the cell necessitates its excretion through specialised copper transporter proteins. ATP7A and ATP7B are pivotal in the extracellular transport of copper (La Fontaine and Mercer, 2007; Shao et al., 2023; Li et al., 2024). ATP7A and ATP7B facilitate the transfer of copper from the trans-golgi network to post-golgi vesicles. These copper-rich vesicles can merge with the plasma membrane and discharge copper into the extracellular space (Xue et al., 2023; Horn and Wittung-Stafshede, 2021). Recent findings indicate that ATP7A functions as a copper exporter, which is increased by mutant KRAS, and that both ATP7A and ATP7B facilitate the chelation and efflux of cisplatin from cells. According to these findings, tumour therapies aimed against ATP7A/B have achieved progress in specific tumours (Aubert et al., 2020; Petruzzelli and Polishchuk, 2019; Arnesano et al., 2011; Lukanović et al., 2020).

## 3 The role of copper in HCC

Copper exhibits a dual role in HCC (Wu et al., 2023), both promoting and inhibiting tumor progression. Elevated copper levels are essential for HCC cell growth and metastasis, a process termed cuproplasia (Zhang et al., 2025). Copper-dependent mechanisms, such as enhanced mitochondrial respiration and activation of pro-angiogenic factors, drive tumor development. However, excessive copper can induce a novel form of cell death called cuproptosis, distinct from apoptosis and necroptosis (Pan et al., 2025). This dual nature of copper underscores the importance of maintaining an optimal copper balance in the tumor microenvironment for effective HCC treatment.

### 3.1 Tumor-stimulative effects of copper in HCC

“Cuproplasia” refers to the function of copper in facilitating cellular growth and proliferation related to hyperplasia, chemotaxis, and tumorigenesis (Ge et al., 2022). It involves copper’s influence on a range of enzyme activities, as well as complex signaling pathways. Maternal embryonic leucine zipper kinase (MELK) has been demonstrated to elevate the expression of the copper death-related gene dihydrolipoamide s-acetyltransferase (DLAT), particularly the ratio of DLAT monomers, through the activation of the PI3K/mTOR pathway. This mechanism can facilitate elesclomol resistance, alter mitochondrial function, and eventually advance HCC progression (Li et al., 2023). Simultaneously, elevated STEAP2 levels are observed in hepatocellular carcinoma, where STEAP2 facilitates HCC cell motility and invasion by enhancing copper levels and activating certain proteins (Torrez et al., 2024).

#### 3.1.1 Copper-mediated oncogenic signaling and energy metabolism

Copper is crucial for the activation of HCC oncogenic signaling pathways. Copper is integral to various critical carcinogenic signaling pathways, including the RAS-RAF-MEK-ERK and PI3K-AKT-mTOR cascades. Copper enhances cancer cell proliferation, viability, and metabolic activity by activating these mechanisms (Asati et al., 2016). Copper serves as a cofactor for mitogen-activated protein kinase kinase 1 (MEK1) and mitogen-activated protein kinase kinase 2 (MEK2), augmenting their influence on the phosphorylation of extra cellular-signal-regulated kinases 1 (ERK1) and extra cellular-signal-regulated kinases 2 (ERK2), thereby activating these signaling pathways. Inhibitors that target specific mutant proteins in the RAS-RAF-MEK1/2-ERK1/2 pathway have been approved for the treatment of malignancies, including HCC (Mandal et al., 2016). This advancement will more efficiently suppress the constitutive activation of this pathway in HCC.

Copper has demonstrated the capacity to enhance energy supply. Cancer cells are distinguished by their accelerated division and proliferation. Furthermore, as copper is vital for ATP synthesis, cancer cells in HCC patients necessitate elevated copper levels compared to non-cancerous cells to fulfil their energy requirements. Copper is a crucial trace element in the electron transport chain of mitochondrial respiration. Copper serves as a cofactor for the essential enzymes mitochondrially encoded cytochrome c oxidase 1 (MT-CO1) and mitochondrially encoded cytochrome c oxidase 2 (MT-CO2) within the mitochondrial respiratory chain, facilitating the electron transport process (Jiang et al., 2021). These two enzymes are critical constituents of cytochrome c oxidase (Complex IV). Additionally, they facilitate the movement of electrons from cytochrome c to oxygen, resulting in the production of water and the release of energy. The roles of copper underscore its significance in HCC formation and establish a theoretical foundation for formulating treatment strategies that target these pathways and energy supply systems.

#### 3.1.2 Copper-dependent cancer cell survival, proliferation and metastasis

Numerous documents indicate that copper promotes cellular autophagy. Copper facilitates the assembly of the autophagy machinery and enhances the survival, development, and proliferation of cancer cells. Copper serves as a cofactor for unc-51-like autophagy-activating kinase (ULK) (Xue et al., 2023), which stimulates autophagosome formation. This autophagic mechanism is also observed in HCC cells and significantly contributes to cancer cell survival, proliferation, and metastasis. Copper facilitates the assembly of autophagic machinery and the creation of autophagosomes, hence offering a crucial mechanism for the survival and proliferation of HCC cells. In conditions of food or energy scarcity, autophagy enables cancer cells to reutilise resources and sustain essential life functions.

Copper is significant for augmenting transcription factor activity in hepatocellular carcinoma progression. Metal regulatory transcription factor-1 (MTF-1) regulates HCC carcinogenesis and progression. Copper exposure markedly promotes HCC cell proliferation by augmenting MTF-1 expression (Lyu et al., 2021). Recent evidence indicates that the selectively targeted exosome miR-148a-3p, functioning as a tumour suppressor across several malignancies (Feng et al., 2020a), may have a role in the negative regulation of MTF-1 in HCC, suggesting therapeutic advantages for HCC patients (Lyu et al., 2021). Hypoxia-inducible factor 1 subunit alpha (HIF1α) is a constituent of a pathway regulating cellular metabolism, and copper stabilises and amplifies HIF1α activity (Feng et al., 2020b). In HCC, increased activity of HIF1α may facilitate cancer cell adaptability and survival, particularly in hypoxic microenvironments. The interferon-induced protein with tetratricopeptide repeats 3 (IFIT3) is recognised for augmenting HCC production by improving interferon-alpha (IFN α) effector signaling, hence promoting IFN α effector responses and treatment efficacy (Yang et al., 2017).

Copper has been demonstrated to facilitate metastasis in hepatocellular cancer. LOX and lysyl oxidase-like protein (LOXL), both copper-dependent enzymes, facilitate tumour invasion and metastasis in cancer by catalysing the cross-linking of extracellular matrix proteins and activating signaling pathways (Rodriguez-Pascual and Rosell-Garcia, 2018). There is growing evidence that the elevation of LOX levels serves as a prediction indicator for HCC and highlights the crucial function of LOX family members in HCC pathogenesis and the modulation of the tumour microenvironment (TME) (Lin et al., 2020). Currently, therapeutic medicines targeting LOX family members for pancreatic and colorectal adenocarcinomas are in the preliminary phases of clinical studies (Wen et al., 2020). Nonetheless, evidence from ClinicalTrials.gov (https://clinicaltrials.gov/ct2/home) indicates a deficiency of clinical trials aimed at LOX family members for the treatment of hepatocellular carcinoma. It indicates that additional investigation into LOX treatment for HCC is necessary.

#### 3.1.3 Copper-induced modulation of tumor proteins

Copper disrupts tumor suppressor function. The tumor protein p53 is a key metabolic regulator that inhibits glycolysis and drives a metabolic shift toward oxidative phosphorylation (Xiong et al., 2023). It has now been shown that copper overload displaces Zn^2+^ from p53, inactivating tumor suppression (Formigari et al., 2013). In addition, intracellular free zinc regulates p53 activity and stability, and copper can displace zinc in the tumor suppressor protein p53, leading to abnormal protein folding and disruption of p53 function (Formigari et al., 2013). A comprehensive examination of copper’s influence on p53 and associated metabolic pathways is anticipated to yield novel insights and strategies for cancer prevention and treatment, hence enhancing the prognosis for cancer patients.

In addition, copper can activate specific degradation pathways. For example, the ubiquitin-proteasome system (UPS) plays a role in maintaining copper homeostasis (Xiong et al., 2023), and copper complexes can inhibit the core components of the UPS. The UPS is a proteolytic metabolism mechanism (Zhang and Burke, 2023), and copper complexes can inhibit the core components of the UPS (Chen et al., 2021). Dihydrolipoamide dehydrogenase (E3) is a key enzyme in the UPS, containing a variety of subfamily proteins involved in the regulation of some common signaling pathways in HCC. Dysregulation of UPS leads to cancer progression, and overexpression of E3 ligases is often associated with poor prognosis. In current cancer treatment studies, the copper complex has been suggested to have the potential as an inhibitor of UPS (Chen et al., 2021). It contributes to its anti-cancer activity and may be a potential target for future HCC treatment strategies.

#### 3.1.4 Copper’s role in tumor inflammation and immune evasion

Copper exacerbates macrophage-mediated inflammation. The mitochondrial reservoir of copper can generate macrophages with an inflammatory phenotype (Chen et al., 2023), which may promote HCC progression. Copper also activates the NF-κB signaling pathway, and NF-κB activation promotes the expression of a series of inflammation-related genes (Hoesel and Schmid, 2013). These genes are crucial in the inflammatory milieu of HCC, resulting in heightened expression of angiogenesis, inflammation, and metastasis-related genes, and provide a possible therapeutic target for HCC.

At the same time, copper also promotes immune escape. Elevated copper levels can increase the expression of the immune checkpoint protein programmed death-ligand 1 (PD-L1), impair anti-tumor immunity, promote cancer immune escape (Voli et al., 2020), and raise the possibility of repurposing copper chelators as anti-tumor immune enhancers. At present, studies have shown that copper chelators can significantly increase the number of cluster of differentiation 8 (CD8) positive T cells and natural killer cells (NK cells) infiltrated by tumors to slow down tumor growth (Voli et al., 2020). It also triggers apoptosis in myeloid-derived suppressor cells (MDSCs) and diminishes their quantity in the tumour microenvironment, hence augmenting the immune response (Zhang S. et al., 2024). The copper-dependent amine oxidase LOXL2 has also been found to be positively correlated with immune cell infiltration and immune checkpoint expression, especially PD-L1 (Radić et al., 2023), which may play a role in predicting the immune response to liver cancer immunotherapy and has become a promising therapeutic target in HCC.

### 3.2 Tumor-suppressive effects of copper in HCC

Copper has a dual role in cancer development. On the one hand, elevated copper levels can promote tumor growth by inducing ROS production, exacerbating genomic instability, and affecting various tumor-related signal transduction events. On the other hand, excessive copper concentration can induce tumor cell death when it exceeds a specific threshold limit (Xue et al., 2023). At present, researchers have found that tumor growth can be effectively inhibited by increasing the concentration of intracellular Cu^+^ or I^−^. They have also developed a Cu-iodine nanoparticle (Cu-I@BSA) targeting mitochondria, which uses the reaction of Cu^+^ and I^−^ to form stable bovine serum albumin (BSA) radiation-induced fluorophores. Under X-ray irradiation, tumor cells are killed, and their energy production and DNA are damaged, promoting cell death (Ma et al., 2024).

#### 3.2.1 Copper-mediated anti-tumor immunity regulation

As an essential trace element for maintaining immune homeostasis, copper influences the tumor microenvironment by regulating immune cells and checkpoints. Currently, immune checkpoint inhibitors such as atezolizumab combined with bevacizumab and tremelimumab combined with durvalumab have improved the survival of HCC patients to some extent (Porcu et al., 2018). Research indicates that the combination therapy of DSF and copper causes immunogenic cell death, augments tumour immunogenicity, and enhances the effectiveness of CD47 inhibition (Gao et al., 2022). Furthermore, the combined therapy of DSF/Cu and anti-PD-1 antibody demonstrated enhanced anti-tumor efficacy compared to monotherapy. The mechanism may involve the upregulation of PD-L1 expression, hence augmenting the therapeutic impact (Zhou et al., 2019). These findings offer novel insights for the utilization of DSF/Cu in the management of HCC.

Recent studies have shown that the expression level of copper transporter ATP7A in HCC is positively correlated with immune cell infiltration and immune checkpoint expression, especially with PD-L1. In addition, HCC patients with high ATP7A expression have higher sensitivity to sorafenib, suggesting that ATP7A may serve as a biomarker to predict response to sorafenib treatment in HCC patients (Shao et al., 2023). This discovery initiates a novel avenue for investigating the function of copper and immune system modulation in hepatocellular carcinoma therapy.

#### 3.2.2 Copper-induced cell death mechanisms

**FIGURE 2:**
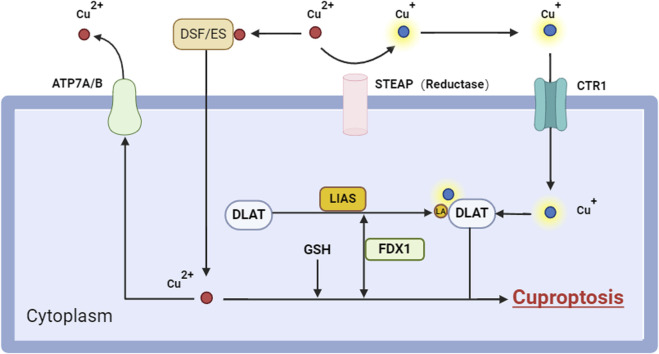
Cuproptosis signaling pathways. Cuproptosis is a type of cellular death, induced by the accumulation of intracellular Cu^2+^, including various critical components and mechanisms. Initially, Cu^+^ infiltrates the cell by copper ionophores (e.g., ES) or copper transporters (e.g., CTR1), which suppress GSH production and liberate Cu ions, resulting in an elevation of intracellular Cu ion concentration. In the cell, Cu ions are converted to the more hazardous Cu^+^ by FDX1, a process that generates reactive oxygen species (ROS). Cu^+^ interacts with lipoacylated proteins like DLAT in mitochondria, facilitating their polymerization and concurrently diminishing the stability of Fe-S cluster proteins. Moreover, the accumulation of copper ions may diminish the release of copper ions by suppressing ATP7B production, hence elevating intracellular copper ion concentrations. Collectively, these activities result in mitochondrial proteotoxic stress that ultimately induces Cuproptosis. Abbreviation: ATP7A/B, ATPase copper taransporting alpha/beta; CTR1, copper transporter 1; DLAT, dihydrolipoamide s-acetyltransferase; DSF, disulfram; ES, elesclomol; FDX1, ferredoxin 1; GSH, glutathione; LA, lipoic acid; LIAS, lipoic acid synthetase; ROS, reactive oxygen species.

Cuproptosis is a form of cell death triggered by copper-induced mitochondrial stress and damage. And it represents a new type of cell death that is intricately associated with copper homeostasis and protein lipoylation (Yang et al., 2025). Tsvetkov et al. first put forward and reported this process and coined it (Tsvetkov et al., 2022). It has been a novel form of programmed cell death, and offered new insights for the research and treatment of hepatocellular carcinoma HCC (Zhu et al., 2025). Unlike other well-known cell death pathways like apoptosis, ferroptosis, pyroptosis, and necroptosis, the induction of cuproptosis typically depends on the proper functioning of copper carriers (Yang et al., 2025). It is characterized by impaired mitochondrial respiration and mitochondrial protein stress (Xie et al., 2023) (Figure 2). DSF/Cu shows considerable cytotoxic effects selectively on HCC cell lines. This phenomenon can significantly disturb mitochondrial equilibrium, elevate the free iron reservoir, and augment lipid peroxidation, finally resulting in ferroptosis. This approach substantially suppressed HCC cell motility, invasion, and angiogenesis (Ren et al., 2021). During cuproptosis, copper interacts with fatty acylated proteins in mitochondria, leading to protein aggregation and cell stress. This abnormal protein aggregation may indirectly affect the function of iron-sulfur tuftin. Iron-sulfur tuftin also plays an important role in ferroptosis, and impairment of its function may trigger the stress response associated with iron death. Therefore, DSF/Cu-induced copper death may be associated with iron death by disrupting the function of iron-sulfur tuftin (Zhu et al., 2024). The cuproptosis-associated gene DLAT exhibited an inverse correlation with overall survival (OS) in HCC patients (Zhou et al., 2022) and influenced cellular metabolism, tumour advancement, and immune system modulation (Zhang et al., 2023). Consequently, it possesses the potential to serve as a novel prognostic biomarker for HCC.

Furthermore, an organometallic compound comprising Cu^2+^, namely, the Cu^2+^ salicylate phenanthroline complex [Cu(sal)(phen)], has been demonstrated to be linked to copper-induced cytotoxicity. This compound demonstrated substantial anti-tumor efficacy against HCC, suppressing the growth of HCC cells (e.g., HepG2 and HCC-LM9) and causing apoptosis in a dose-dependent manner (Niu et al., 2023). Furthermore, research utilising machine learning methods and artificial neural networks has demonstrated that LGOd1 promotes apoptosis by disrupting copper homeostasis in HCC cells, potentially representing a novel class of drugs with distinctive copper-inducing characteristics (Yang et al., 2024).

Research on the potential role of the cuproptosis pathway in HCC is becoming increasingly in-depth. The current study shows that the copper death-dependent protein ferredoxin (FDX) plays a significant role in HCC: disruption of FDX1 promotes tumor cell proliferation and migration, whereas high expression of FDX2 reduces cell viability in HCC samples (Quan et al., 2023). These findings may provide new strategies for the treatment of HCC, especially in regulating the tumor immune microenvironment and influencing patient prognosis.

#### 3.2.3 Copper-dependent tumor-suppressive signaling modulation

Previous studies have shown that the Copper metabolism gene MURR1 domain 10 (COMMD10), which regulates intracellular copper balance and distribution, can inhibit the proliferation of HCC cells and induce apoptosis by inhibiting NF-κB signal transduction (Yang et al., 2021). In addition, COMMD10 was closely correlated with barcelona clinic liver cancer (BCLC) staging in predicting OS, providing important evidence for identifying potential therapeutic targets and accurately predicting prognosis in patients with liver cancer. Additional research has verified that COMMD10 may enhance copper synthesis during ionising radiation (IR), resulting in resistance to radiotherapy (Yang M. et al., 2022), so presenting a novel therapeutic target for augmenting the sensitivity of HCC to radiotherapy. The diminished expression of the Copper metabolism gene MURR1 domain 3 (COMMD3) can impede the angiogenesis of HCC by obstructing the HIF1α/VEGF/NF-κB pathway (Zhu et al., 2022; Cheng et al., 2022). It suggests that COMMD3 may be a potential biomarker to improve the therapeutic efficacy of HCC (Zhu et al., 2022; Wang et al., 2021b).

#### 3.2.4 Copper chelators and copper ionophore

Copper chelators, which are pharmacological agents that specifically target copper to reduce its ion concentration in the body, are recognized as a prospective anti-cancer treatment approach. They function by obstructing the pro-survival effects of copper in neoplastic cells and play a significant role in cancer treatment. Presently, prevalent copper chelators comprise Tetrathiomolybdate (TTM) and Trientine. TTM is a copper chelator that can inhibit copper absorption, thereby reducing the tumorigenicity of HCC cell lines (Zhang et al., 2025). It also inhibits glycolysis, reducing the energy supply to tumor cells, and thereby impeding tumor initiation and progression (Kirk et al., 2024). TTM may restrict ATP synthesis in tumour cells by obstructing the mitochondrial tricarboxylic acid cycle and glycolysis and it inhibits tumor growth and angiogenesis by chelating copper (Ishida et al., 2013b; Liu et al., 2021). Conversely, Trientine promotes apoptosis by activating the P38 mitogen-activated protein kinase and suppresses endothelial cells in HCC as well as angiogenesis, hence restricting tumour proliferation (Yang Z. et al., 2022). Trientine can deplete copper levels in HCC cells, thereby inhibiting angiogenesis and tumor growth (Yin et al., 2016; Yoshiji et al., 2003). It has been shown to reduce copper-dependent processes that drive tumor progression and induce apoptosis in HCC cells (Zhou et al., 2023). The main mechanism of action of trientine is to reduce the bioavailability of copper through the copper in the chelate. Copper plays an important role in tumor growth and angiogenesis, so reducing copper levels can inhibit tumor development (Zhou et al., 2023).

Copper ionophore offers a novel approach to specifically target neoplastic cells. Elesclomol, a copper ionophore utilised in HCC treatment, facilitates copper influx into cells and induces copper-mediated cytotoxicity, hence impeding HCC progression. DSF/Cu induces endoplasmic reticulum stress, disrupts endoplasmic reticulum calcium equilibrium, and ultimately results in copin-induced cellular apoptosis (Zhang P. et al., 2024). DSF/Cu effectively downregulated the expression of the PTEN/Akt signaling pathway, consequently decreasing the viability and proliferation of HCC cells due to cellular oxidative stress. The identification of these mechanisms enhances our comprehension of copper’s function in tumour biology and offers significant guidance for the creation of innovative anti-cancer pharmaceuticals.

#### 3.2.5 Latest copper-based HCC therapeutic strategies

Sorafenib is presently an efficacious first-line treatment for advanced HCC (Tang et al., 2020). Consequently, owing to its inadequate water solubility, researchers engineered an innovative copper-based metal-organic framework (MOF) nanocatalyst. It integrates the cytochrome c oxidase copper chaperone 2 (COX2) inhibitor meloxicam with the chemotherapeutic agent sorafenib to augment the therapeutic efficacy against HCC via a cascade reaction (Tian et al., 2022).

Additionally, research has demonstrated that the synergistic effects of certain pharmaceuticals can significantly enhance cancer therapy. The combination of 5-FU with DSF demonstrated substantial enhancements in the majority of measures over the treatment period. The 5-FU-Cu combination treatment group induced apoptosis in cancer cells to a considerable degree (Hassan et al., 2023). The combination of DSF/Cu^+^ with sorafenib demonstrated more efficacy than sorafenib alone in enhancing autophagy and apoptosis in HCC cells (Li et al., 2021). These trials validated the possibility of medication synergism in enhancing therapeutic efficacy. Simultaneously, they proposed that greater emphasis be placed on the investigation of medication combinations in forthcoming HCC treatment.

Moreover, researchers are diligently investigating synergistic therapies among various therapy modalities. In the most recent study, researchers created a tumour microenvironment stimulus-responsive nanomedicine delivery system utilising UCCu^2+^NPs for the treatment of HCC. It employs the introduction of Cu^2+^ to facilitate charge inversion and lysosomal escape using a triad method of chemodynamic, phototherapeutic, and heat-enhanced chemodynamic therapy (CDT) (Lai et al., 2023). Lenvatinib (LT), increasingly supplanting sorafenib as a targeted therapy for advanced HCC, has also been shown to function as a chemotherapeutic agent in photothermal therapy utilising an innovative biophotonic nanoplatform composed of copper sulphide nanocrystals with near-infrared (NIR) photothermal characteristics. It surpasses the efficacy of any individual therapeutic or theoretical amalgamation (Xu et al., 2021; Xu et al., 2023). With the development of these innovative therapies, we are more hopeful about the comprehensive treatment strategy for HCC.

64CuCl2 is an efficient positron emission tomography (PET) radiotracer for the diagnosis of HCC, utilising the elevated copper uptake characteristic of HCC. It holds considerable importance for HCC diagnostic imaging and offers a novel therapeutic avenue for radionuclide treatment of HCC (Peng, 2022). Furthermore, Doxorubicin(DOX)@BSA-CuS, a CuS nanotherapeutic agent utilising DOX encapsulation and NIR responsiveness, exhibited substantial tumour growth inhibition and indicated clinical translational promise in imaging-guided arterial embolisation therapy for HCC (Li et al., 2021). These advancements indicate that copper-based therapy approaches possess significant potential and applicability in the diagnosis and treatment of HCC.

## 4 Conclusion

This study demonstrated the significant involvement of copper in hepatocellular carcinoma and its correlation with disease advancement. It elucidates its intricate regulatory processes in the progression of hepatocellular carcinoma. The dysregulation of copper metabolism is intricately linked to the advancement of HCC. A comprehensive understanding of the molecular mechanisms governing its transit and interaction with HCC cells is essential for attaining effective treatment. Despite copper’s essential function in numerous cellular activities, our comprehension of its molecular action mechanism remains inadequate.

To enhance copper-targeted therapy, it is essential to develop strategies for modulating copper levels or targeting copper metabolic signaling pathways in patients with HCC. The advancement of techniques to precisely measure total copper concentrations in cells and plasma will facilitate the identification of patient cohorts likely to benefit from copper-targeted medicines and the creation of suitable companion diagnostic instruments. Simultaneously, measuring a patient’s “copper status” is crucial for enhancing treatment, reducing adverse effects, and assessing effectiveness.

The primary issue is to enhance the specificity of treatments for hepatocellular carcinoma that target aberrant copper metabolism. We anticipate instilling new hope in patients by enhancing our comprehension of copper homeostasis mechanisms and converting this insight into useful therapy options for HCC. As our study advances, we anticipate closing the gap between copper homeostasis and HCC treatment, thereby offering more precise and effective therapeutic choices for liver cancer patients.

## Glossary

ATOX1: antioxidant 1 copper chaperone
ATP: adenosine triphosphate
ATP7A: ATPase copper taransporting alpha
ATP7B: ATPase copper transporting beta
BCLC: barcelona clinic liver cancer
BSA: bovine serum albumin
CCO: cytochrome c oxidase
CCS: copper chaperone for superoxide dismutase
CD44: cluster of differentiation 44
CD8: cluster of differentiation 8
CDT: chemodynamic therapy
COMMD10: copper metabolism gene MURR1 domain 10
COMMD3: copper metabolism gene MURR1 domain 3
Complex IV: cytochrome c oxidase
COX17: cytochrome c oxidase copper chaperone 17
COX2: cytochrome c oxidase copper chaperone 2
CP: ceruloplasmin
CTR1: copper transporter 1
CTR2: copper transporter 2
Cu-I@BSA: cu-iodine nanoparticle
DLAT: dihydrolipoamide s-acetyltransferase
DMT1: divalent metal-ion transporter-1
DOX: doxorubicin
DRP1: dynamin - related protein 1
DSF: disulfiram.E3, dihydrolipoamide dehydrogenase
ERK1: extra cellular-signal-regulated kinases 1
ERK2: extra cellular-signal-regulated kinases 2
FDX: ferredoxin
GSH: glutathione
HCC: hepatocellular carcinoma
HIF1: hypoxia inducible factor 1
HIF1α: hypoxia inducible factor 1 subunit alpha
HUVEC: human umbilical vein endothelial cell
IFIT3: interferon-induced protein with tetratricopeptide repeats 3
IFN α: interferon-alpha
IR: ionising radiation
LOX: lysyl oxidase
LOXL: lysyl oxidase - like protein
LT: Lenvatinib
MDSCs: myeloid-derived suppressor cells
MEK1: mitogen-activated proteinkinase kinase 1
MEK2: mitogen-activated proteinkinase kinase 2
MELK: maternal embryonic leucine zipper kinase
MEMO1: the protein mediator of ERBB2-driven cell motility 1
MOF: copper-based metal-organic framework
MT: metallothionein
MT-CO1: mitochondrially encoded cytochrome c oxidase 1
MT-CO2: mitochondrially encoded cytochrome c oxidase 2
MTF-1: metal regulatory transcription factor-1
NIR: near-infrared window
NK: cells, natural killer cell
OS: overall survival
PD-L1: programmed death-ligand 1
PET: position emission tomography
ROS: reactive oxygen species
SLC25A3: solute carrier family 25 member 3
SOD1: superoxide dismutase 1
TGN: trans golgi network
TTM: tetrathiomolybdate
TME: tumour microenvironment
ULK: unc-51-like autophagy-activating kinase
UPS: ubiquitin-proteasome system.
